# Involvement of ABA Responsive *SVB* Genes in the Regulation of Trichome Formation in Arabidopsis

**DOI:** 10.3390/ijms22136790

**Published:** 2021-06-24

**Authors:** Saddam Hussain, Na Zhang, Wei Wang, Sajjad Ahmed, Yuxin Cheng, Siyu Chen, Xutong Wang, Yating Wang, Xiaojun Hu, Tianya Wang, Shucai Wang

**Affiliations:** 1 Key Laboratory of Molecular Epigenetics of MOE, School of Life Sciences, Northeast Normal University, Changchun 130024, China; hase705@nenu.edu.cn (S.H.); zhangn906@nenu.edu.cn (N.Z.); wangw716@nenu.edu.cn (W.W.); ahmed80@yorku.ca (S.A.); chengyx104@nenu.edu.cn (Y.C.); chensy564@nenu.edu.cn (S.C.); wangxt357@nenu.edu.cn (X.W.); wangyt814@nenu.edu.cn (Y.W.); wangty309@nenu.edu.cn (T.W.); 2 Laboratory of Plant Molecular Genetics & Crop Gene Editing, School of Life Sciences, Linyi University, Linyi 276000, China; huxiaojun@lyu.edu.cn

**Keywords:** trichome formation, ABA, SVB, SVB2, transcription factor, CRISPR/Cas9 gene editing, Arabidopsis

## Abstract

Trichome formation in Arabidopsis is regulated by several key regulators, and plants hormones such as gibberellin, salicylic acid, jasmonic acid and cytokinins have been shown to regulate trichome formation by affecting the transcription or activities of the key regulators. We report here the identification of two abscisic acid (ABA) responsive genes, *SMALLER TRICHOMES WITH VARIABLE BRANCHES* (*SVB*) and *SVB2* as trichome formation regulator genes in Arabidopsis. The expression levels of *SVB* and *SVB2* were increased in response to ABA treatment, their expression levels were reduced in the ABA biosynthesis mutant *aba1-5*, and they have similar expression pattern. In addition to the trichome defects reported previously for the *svb* single mutant, we found that even though the trichome numbers were largely unaffected in both the *svb* and *svb2* single mutants generate by using CRISPR/Cas9 gene editing, the trichome numbers were greatly reduced in the *svb svb2* double mutants. On the other hand, trichome numbers were increased in *SVB* or *SVB2* overexpression plants. RT-PCR results show that the expression of the trichome formation key regulator gene *ENHANCER OF GLABRA3* (*EGL3*) was affected in the *svb svb2* double mutants. Our results suggest that *SVB* and *SVB2* are ABA responsive genes, and SVB and SVB2 function redundantly to regulate trichome formation in Arabidopsis.

## 1. Introduction

Trichomes are developed from epidermal cells on the surface of the plant aerial parts, and they can protect plants from some of the biotic and abiotic stresses such as excessive heat, water loss, and insect or pathogen attacks, due to their ability to increase thickness of the boundary layer between epidermal surface and environment [1,2].

As a good model for studying cell fate determination, trichome formation in Arabidopsis has been extensively studied. Accumulated evidence suggests that the key regulators of trichome formation in Arabidopsis are a few transcription factors [3,4,5,6,7]. These transcription factors including the WD40-repeat protein TRANSPARENT TESTA GLABRA1 (TTG1) [8], the R2R3 MYB transcription factor GLABRA1 (GL1) [9], the bHLH transcription factor GLABRA3 (GL3) or ENHANCER OF GLABRA3 (EGL3) [10,11], the homeodomain protein GLABRA2 (GL2) [12], and the R3 MYB transcription factors including TRYPTICHON (TRY), CAPRICE (CPC), ENHANCER OF TRY AND CPC1 (ETC1), ETC1, ETC3, TRICHOMELESS1 (TCL1) and TCL2 [13,14,15,16,17,18,19,20,21].

TTG1, GL1 and GL3/EGL3 are able to form a MYB-bHLH-WD40 (MBW) complex to activate the expression of *GL2*, therefore promote trichome formation [3,4,5,6,7]. This MBW complex is also able to activate the expression of some R3 MYB genes including *TRY*, *CPC*, *ETC1* and *ETC3* [13,14,15,17,18,20]. These R3 MYB proteins including ETC2, TCL1 and TCL2 can move to the neighboring cells, where they compete with GL1 for binding GL3, therefore blocking the formation of the MBW complex, hence resulting in the inhibition of trichome formation [3,4,5,6,7,22,23,24].

In addition to the key regulators, several other types of transcription factors have been found to regulate trichome formation in Arabidopsis, by regulating gene expression and/or the activities of the key regulators. For example, the C2H2 transcription factors GLABROUS INFLORESCENCE STEMS (GIS) and GIS3, and the ZINC FINGER transcription factors ZINC FINGER PROTEIN 5 (ZFP5) and ZFP8 regulate the expression of the MBW complex component genes [25,26,27,28,29], the plant-specific transcription factor SQUAMOSA PROMOTER BINDING PROTEIN LIKE (SPL) transcription factor SPL9 and the NAM, ATAF1/2, and CUC (NAC) transcription factor NTM1-LIKE 8 (NTL8) directly regulates the expression of R3 MYB genes *TRY* and *TCL1* [30,31], whereas the CINCINNATA-like TEOSINTE BRANCHED1-CYCLOIDEA-PCF (TCP) transcription factor TCP4 directly regulate the expression of R3 MYB genes *TCL1* and *TCL2* [32]. On the other hand, the TCP proteins such as TCP2, TCP3, TCP4, TCP5, TCP10, TCP13, TCP17 and TCP24 can interact directly with GL3, therefore affecting the formation of the MBW complex [33].

It should be noted that the plant hormone gibberellin (GA) is able to regulate the expression of *ZFP6*, and cytokinins (CTK) is able to regulate the expression of *ZFP8* and *GIS2* [25,26,28,29], therefore are involved in the regulation of trichome formation. The plant hormone jasmonic acid (JA) is also involved in the regulation of trichome formation in Arabidopsis. The Jasmonate ZIM-domain (JAZ) proteins, the key negative regulators of JA signaling [34,35], are able to interact with GL1, GL3 and EGL3, therefore affecting the formation of the MBW complex [36]. The plant hormone abscisic acid (ABA) is a key stress hormone that regulates plant abiotic stress responses via signal transduction [37,38,39,40,41]. However, it remained unknown whether ABA may also involve in the regulation of trichome formation in Arabidopsis.

ABA signaling through the Pyrabactin resistance 1/PYR1-like/Regulatory component of ABA (PYR1/PYL/RCAR) receptors, the A-group PROTEIN PHOSPHATASE 2C (PP2C) phosphatases, the NONFERMENTING 1 (SNF1)-RELATED PROTEIN KINASES (SnRK) protein kinases, and the ABA-RESPONSIVE ELEMENT BINDING FACTOR/ABA-RESPONSIVE ELEMENT BINDING PROTEIN/ABA INSENSITIVE 5 (ABF/AREB/ABI5)-type bZIP (basic region leucine zipper) transcription factors results in the activation or repression of hundreds and thousands of ABA responsive genes [38,41,42,43,44,45,46]. However, functions of most ABA responsive genes remained unknown.

SMALLER TRICHOMES WITH VARIABLE BRANCHES (SVB), a DUF538 domain containing protein was initially identified as a regulator of trichome morphology in Arabidopsis, and *svb* mutant produced small trichomes with variable branches [47], and expression of *SVB* under its native promoter recovered the trichome phenotypes in the *svb* mutant [48]. SVB was then identified as a PI(3)P and PI(3,5) P2 binding protein, and salt affects the binding of SVB with PI(3)P and PI(3,5) P2 [49]. Recently, it has been shown that the expression of *SVB* is induced by tunicamycin-induced ER stress, and SVB is required for ER stress tolerance [48].

In an attempt to identify novel plays in ABA signaling by exploring available transcriptome dataset [50], we found the expression of *SVB* was highly induced by ABA, with an RPKM of 174 in control compared to 846.9 in ABA treated samples, indicating that ABA may play a role in regulating trichome morphology and/or trichome formation. Here we report the identification of both *SVB* and its closest related DUF538 gene, *SVB2* as ABA responsive genes, and we show that SVB and SVB2 function redundantly to regulate trichome formation in Arabidopsis via affecting the expression of some trichome formation key regulator genes.

## 2. Results

### 2.1. Expression of SVBs Are Regulated by ABA

**Figure 1:**
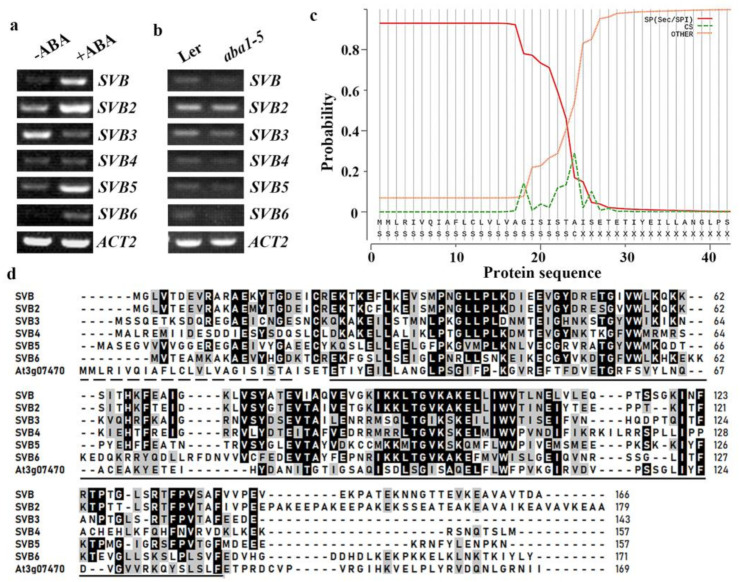
*SVB* is an ABA response gene and is closely related to other 5 *SVBs*. (**a**) Expression of *SVBs* in response to ABA. Twelve-day-old Col wild type seedlings were treated with 50 μM ABA for 4 h, RNA was isolated, and RT-PCR was used to examine the expression of *SVBs*. The expression of *ACT2* was used as a control. (**b**) Expression of *SVBs* in the ABA biosynthesis mutant *aba1-5*. RNA was isolated from 12-day-old Ler wild type and *aba1-5* mutant seedlings, and RT-PCR was used to examine the expression of *SVBs*. The expression of *ACT2* was used as a control. (**c**) Signaling peptide prediction of At3g07470. The full-length amino acid sequence of At3g07470 was used for signal peptide prediction on SignalP (http://www.cbs.dtu.dk/services/SignalP, accessed on 4 June 2021). (**d**) Amino acid alignment of SVBs and At3g07470. The full-length amino acid sequences of SVBs and At3g07470 were obtained from phytozome (https://phytozome.jgi.doe.gov/pz/portal.html#, accessed on 4 June 2021) and sequence alignment was performed by using BioEdit 7.0 (https://bioedit.software.informer.com/7.0/, accessed on 4 June 2021). The identical amino acids were shaded in black, and the similar ones in gray. Solid underline indicates the DUF538 domain. Dash underline indicates the signal peptide in At3g07470.

Available transcriptome dataset indicates that the expression of *SVB* is induced by ABA treatment [50]. To test if this is indeed the case, we examined the expression of *SVB* in response to ABA treatment by using RT-PCR. Col wile type Arabidopsis seedlings were treated with ABA, RNA was isolated and subjected to RT-PCR analysis. As shown in Figure 1a, the expression level of *SVB* increased dramatically in Arabidopsis seedlings treated with ABA when compared with that in the control seedlings.

We further examined the expression of *SVB* in seedlings of the ABA biosynthesis mutant *aba1-5* [51], and found that the expression level of *SVB* was decreased in the *aba1-5* mutant when compared with that in the Ler wild type (Figure 1b). These results suggest that *SVB* is an ABA response gene.

It has been reported that there are 5 SVB homologs in Arabidopsis [48]. Protein homologs assays on Phytozome (https://phytozome.jgi.doe.gov/pz/portal.html#, accessed on 4 June 2021) show that SVB indeed shared high similarities with some other DUF538 proteins. Different from SVB and its 5 homologs, At3g07470, the next closely related DUF538 protein, has an N-terminal signal peptide as predicted by SignalP (http://www.cbs.dtu.dk/services/SignalP, accessed on 4 June 2021) (Figure 1c). Amino acid sequence alignment also show that SVB shared high amino acid sequence identity and similarity at the DUF538 domain with its 5 homologs, but not At3g07470 (Figure 1d) These results suggest that there are a total of 6 SVBs in Arabidopsis, therefore we named the 5 SVB homologs SVBs, i.e., SVB2 (At1g09310), SVB3 (At5g46230), SVB4 (At1g30020), SVB5 (At4g24130) and SVB6 (At5g49600).

We therefore examined ABA response of other five *SVB* genes, we found that the expression levels of *SVB2*, *SVB5* and *SVB6* were increased, but the expression level of *SVB3* was decreased in response to ABA, whereas the expression level of *SVB4* remained largely unchanged (Figure 1a). Similar to that of *SVB*, the expression level of *SVB5* and *SVB6* were decreased in the *aba1-5* mutant seedlings (Figure 1b).

### 2.2. SVB and SVB2 Have Similar Expression Pattern and Similar Protein Subcellular Localization

**Figure 2:**
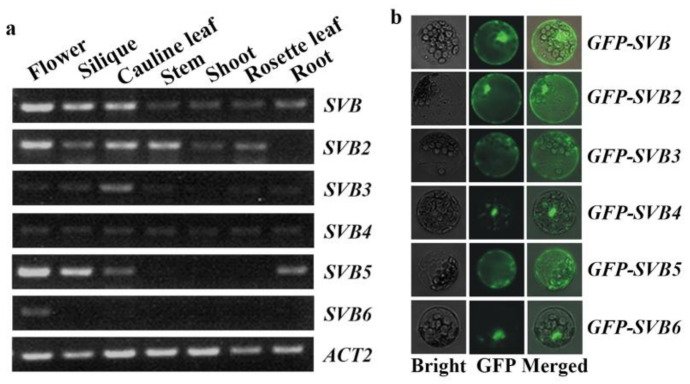
Expression patterns of *SVBs* and subcellular localization of SVBs. (**a**) Expression pattern of *SVBs*. RNA was isolated from different tissues and organs collected from the Col wild type Arabidopsis plant, and RT-PCR was used to examine the expression of *SVBs*. The expression of *ACT2* was used as a control. (**b**) Subcellular localization of SVBs. Plasmids of the effector genes *GFP-SVBs* were transfected into protoplasts isolated from rosette leaves of 3-4 weeks old Col wild type Arabidopsis plants, and GFP fluorescence was observed and photographed under a confocal microscope after the transfected protoplasts were incubated in darkness at room temperature for 20~22 h.

By using *promoter-GUS* transgenic plants, Yu and Kanehara [48] have shown that *SVB* is highly expressed at different development stages. To examine the functions of SVBs, we examined expression pattern of *SVBs*. Different tissues and organs were collected, RNA was isolated, and used for RT-PCR analysis. We found that the *SVBs* showed different expression patterns. *SVB*, *SVB2* and *SVB4* are expressed in all the tissue and organs examined, but the expression pattern are somewhat different. *SVB* and *SVB2* have largely similar expression patterns, with the highest expression levels observed in flowers, whereas *SVB4* is ubiquitously expressed in all the tissues and organs examined (Figure 2a).

On the other hand, *SVB3*, *SVB5* and *SVB6* showed tissue specific expression patterns. *SVB3* is mainly expressed in cauline leaves, *SVB5* is mainly expressed in flowers, siliques, cauline leaves, and roots, whereas *SVB6* is mainly expressed in flower (Figure 2a).

Previously report has shown that SVB is localized in multiple organelles of the cells, including plasma membrane, prevacuolar compartment, Golgi apparatus and endoplasmic reticulum (ER) [48]. By using protoplast transfection assays, we found that GFP florescence was observed all over the cell for SVB, SVB2, SVB3 and SVB5, but SVB4 and SVB6 were predominantly localized in nucleus (Figure 2b).

### 2.3. SVB but Not Other SVBs Affect Trichome Development

**Figure 3:**
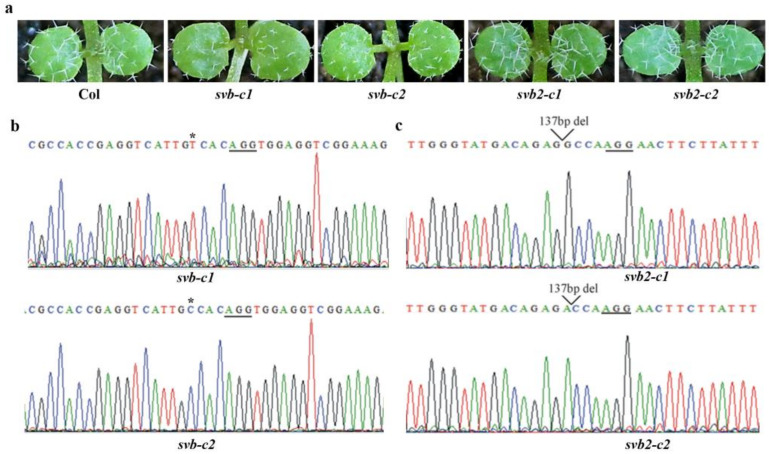
Mutations of *SVB* but not *SVB2* affect trichome development. (**a**) Trichome phenotypes of the Col wild type, the *svb* and *svb2* single mutants. Seeds of the Col wild type, the *svb-c1*, *svb-c2*, *svb2-c1* and *svb2-c2* mutants were sow directly into soil pots and grown in a growth room, and trichome phenotypes on the first two rosette leaves of 10-day-old seedlings were examine under a Motic K microscope and pictures were taken by using an EOS 1100D digital camera connected to the microscope. (**b**) Editing status of *SVB* in the *svb-c1* and *svb-c2* mutants. DNA was isolated from normal flowering T2 plants, and sequenced. Underlines indicate the PAM sites, and stars indicate the single nucleotide inserted in the target sequences. (**c**) Editing status of *SVB2* in the *svb2-c1* and *svb2-c2* mutants. DNA was isolated from normal flowering T2 plants, and sequenced. Underlines indicate the PAM sites, and open arrow heads indicate the sites where small fragments were deleted.

Similar ABA responses and similar expression patterns suggest that SVB and SVB2 may have similar functions. To examine if that is the case, we generated gene edited mutants for *SVB* and *SVB2*, respectively by using CRISPR/Cas9 gene editing. We found that both the *svb-c1* and *svb-c2* mutants produced small trichomes with variable branches (Figure 3a), similar to previously reported for the *svb* T-DNA insertion mutants [47,48]. In both the *svb-c1* and *svb-c2* mutants, only one target was edited, and a single nucleotide was inserted at one of the target sites (Figure 3b). However, unlike that in the *svb* mutants, trichomes in both the *svb2-c1* and *svb2-c2* mutants were largely indistinguishable from that of the Col wild type (Figure 3a). In both the *svb2-c1* and *svb2-c2* mutants, both target sites were edited, resulting in a 137bp fragment deletion (Figure 3c), suggest that these mutants should be loss-of-function mutants.

**Figure 4:**
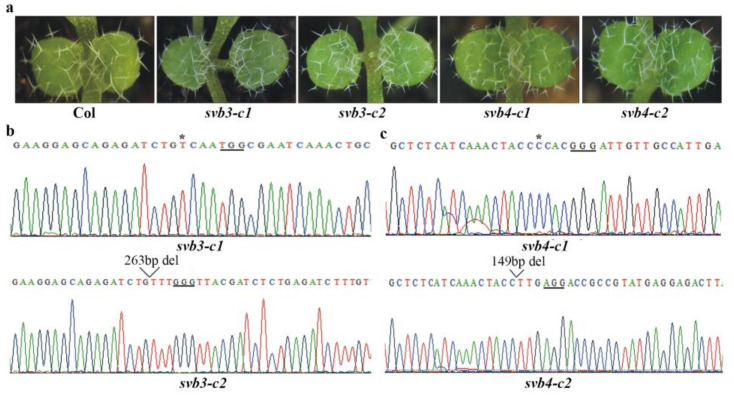
Mutations of *SVB3* or *SVB4* did not affect trichome development. (**a**) Trichome phenotypes of the Col wild type, the *svb3* and *svb4* single mutants. Seeds of the Col wild type, the *svb3-c1*, *svb3-c2*, *svb4-c1* and *svb4-c2* mutants were sown directly into soil pots and grown in a growth room, and trichome phenotypes on the first two rosette leaves of 8-day-old seedlings were examine under a Motic K microscope and pictures were taken by using an EOS 1100D digital camera connected to the microscope. (**b**) Editing status of *SVB3* in the *svb3-c1* and *svb3-c2* mutants. DNA was isolated from normal flowering T2 plants, and sequenced. Underlines indicate the PAM sites, star indicates the single nucleotide insertion in the target sequence, and open arrow head indicates the site where a small fragments was deleted. (**c**) Editing status of *SVB4* in the *svb4-c1* and *svb4-c2* mutants. DNA was isolated from normal flowering T2 plants, and sequenced. Underlines indicate the PAM sites, star indicates the single nucleotide insertion in the target sequence, and open arrow heads indicate the sites where small fragments were deleted.

**Figure 5:**
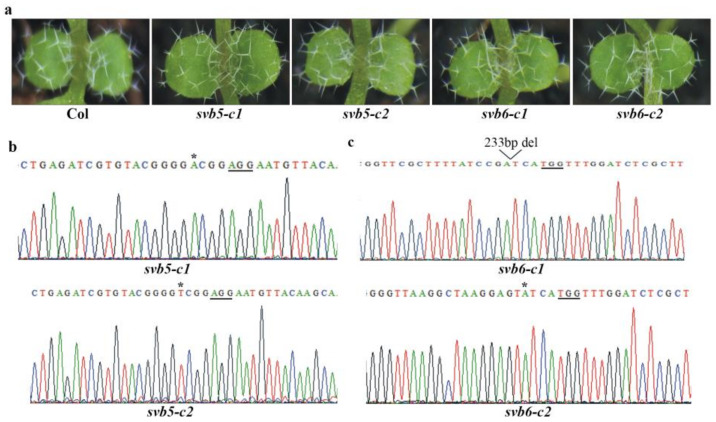
Mutations of *SVB5* or *SVB6* did not affect trichome development. (**a**) Trichome phenotypes of the Col wild type, the *svb5* and *svb6* single mutants. Seeds of the Col wild type, the *svb5-c1*, *svb5-c2*, *svb6-c1* and *svb6-c2* mutants were sown directly into soil pots and grown in a growth room, and trichome phenotypes on the first two rosette leaves of 8-day-old seedlings were examine under a Motic K microscope and pictures were taken by using an EOS 1100D digital camera connected to the microscope. (**b**) Editing status of *SVB5* in the *svb5-c1* and *svb5-c2* mutants. DNA was isolated from normal flowering T2 plants, and sequenced. Underlines indicate the PAM sites, and stars indicate the single nucleotide insertion in the target sequences. (**c**) Editing status of *SVB6* in the *svb6-c1* and *svb6-c2* mutants. DNA was isolated from normal flowering T2 plants, and sequenced. Underlines indicate the PAM sites, star indicates the single nucleotide insertion in the target sequence, and open arrow head indicates the site where a small fragment was deleted.

To exam if other SBVs may also involve in trichome development, we generated single mutants for *SVB3* to *SVB6* genes, as shown in Figure 4a, trichomes in both the *svb3* and *svb4* mutants were largely similar to the Col wild type. In the *svb3* mutants, either one target or both targets were edited, resulting in a single nucleotide insertion and a 263bp fragment deletion, respectively (Figure 4b). Similarly, the *svb4* mutants have either a single nucleotide insertion or a 149bp fragment deletion (Figure 4c). Trichomes in the *svb5* and *svb6* mutants were also largely unaffected (Figure 5a). In both the *svb5* mutants, only one target was edited, resulting in a single nucleotide insertion (Figure 5b), whereas in the *svb6* mutants, either a single nucleotide was inserted or a 233bp fragment was deleted (Figure 5c).

### 2.4. SVB and SVB2 Function Redundantly to Regulate Trichome Formation

**Figure 6:**
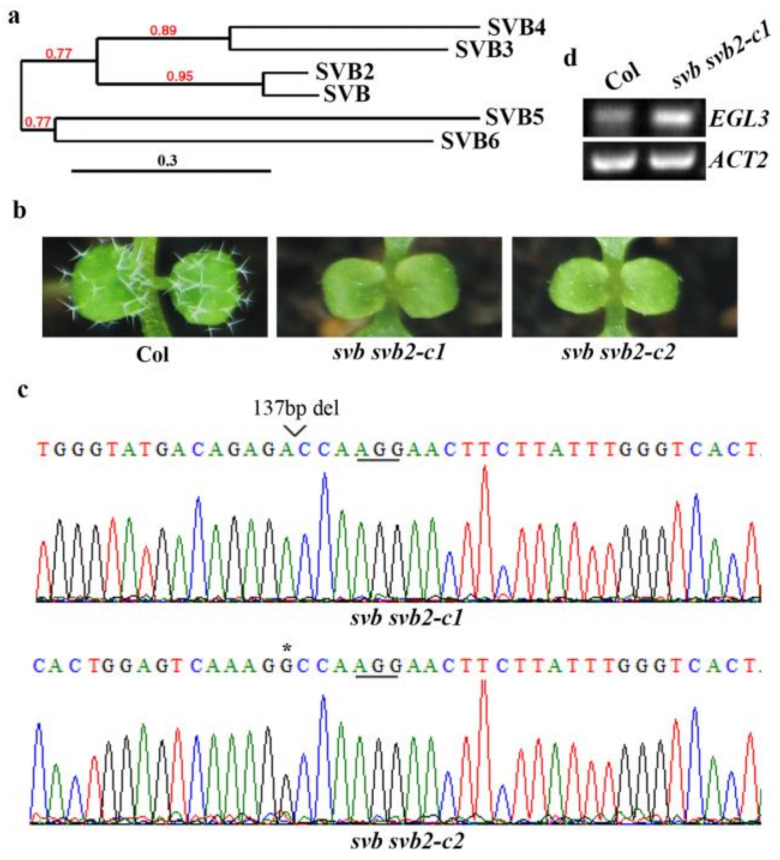
SVB and SVB2 function redundantly to regulate trichome formation. (**a**) Phylogenetic analysis of SVBs. Full-length amino acid sequences of the SVBs were obtained on Phytozome (https://phytozome.jgi.doe.gov/pz/portal.html#, accessed on 4 June 2021), and used for phylogenetic analysis on phylogeny (http://www.phylogeny.fr/simple_phylogeny.cgi, accessed on 4 June 2021) “One Click” mode with default settings was used for the assays. Bar indicates branch length, and numbers above the branches indicate support values. (**b**) Trichome formation on the first two rosette leaves of the Col wild type and the *svb svb2* double mutants. Seeds of the Col wild type, the *svb svb2-c1* and *svb svb2-c2* double mutants were sow directly into soil pots and grown in a growth room, and trichomes on the first two rosette leaves of 10-day-old seedlings were examined under a Motic K microscope and pictures were taken by using an EOS 1100D digital camera connected to the microscope. (**c**) Editing status of *SVB2* in the *svb svb2-c1* and *svb svb2-c2* double mutants. DNA was isolated from normal flowering T2 plants and used for sequencing. Underlines indicate the PAM sites, star indicates the single nucleotide insertion in the target sequence, and open arrow head indicates the site where a small fragment was deleted. (**d**) Expression of *EGL3* in the *svb svb2-c1* double mutants. RNA was isolated from 12-day-old seedlings of the Col wild type and the *svb svb2-c1* double mutants, and RT-PCR was used to examine the expression of *EGL3*. The expression of *ACT2* was used as a control.

The results described above show that *SVB* shared similar ABA response and similar expression pattern with *SVB2*, phylogenic analysis on Phylogeny (www.phylogeny.fr, accessed on 4 June 2021) shows that SVB is closely related to SVB2 (Figure 6a). Therefore, we examined if SVB and SVB2 may function redundantly to regulate trichome development in Arabidopsis.

We therefore generated *svb svb2* double mutants by editing *SVB2* in the *svb-c2* mutant background. We found that both the *svb svb2-c1* and *svb svb2-c2* mutants showed a glabrous-like phenotype, with only smaller trichomes were observed on the rosette leaves of the mutants (Figure 6b). In the double mutants, both target sites of *SVB2* were edited, resulting in a 137bp fragment deletion and a single nucleotide insertion in the *svb svb2-c1* and *svb svb2-c2* mutants, respectively (Figure 6c), suggesting that these mutants should be loss-of-function mutants.

**Figure 7:**
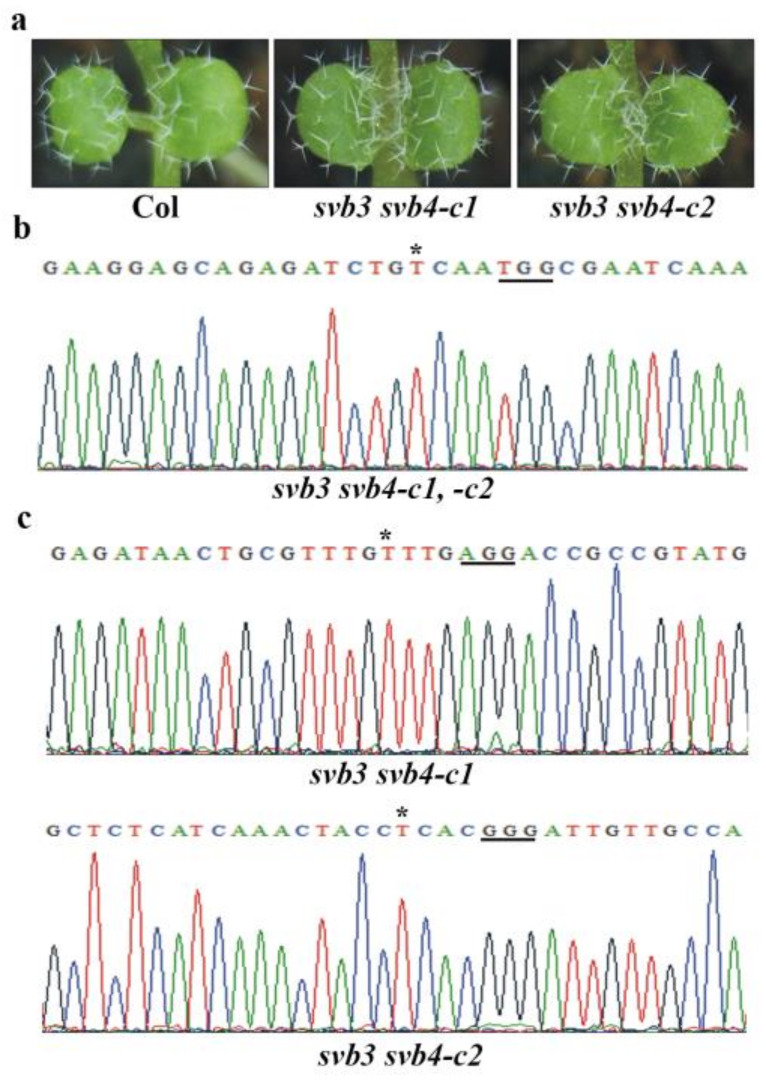
Mutations of *SVB3* and *SVB4* did not affect trichome development. (**a**) Trichome formation on the first two rosette leaves of the Col wild type and the *svb3 svb4* double mutants. Seeds of the Col wild type, the *svb3 svb4-c1* and *svb3 svb4-c2* double mutants were sown directly into soil pots and grown in a growth room, and trichomes on the first two rosette leaves of 8-day-old seedlings were examine under a Motic K microscope and pictures were taken by using an EOS 1100D digital camera connected to the microscope. (**b**) Editing status of *SVB3* in the *svb3 svb4-c1* and *svb3 svb4-c2* double mutants. (**c**) Editing status of *SVB4* in the *svb3 svb4-c1* and *svb3 svb4-c2* double mutants. DNA was isolated from normal flowering T2 plants, and used for sequencing. Underlines indicate the PAM sites, and stars indicate the single nucleotide inserted in the target sequences.

**Figure 8:**
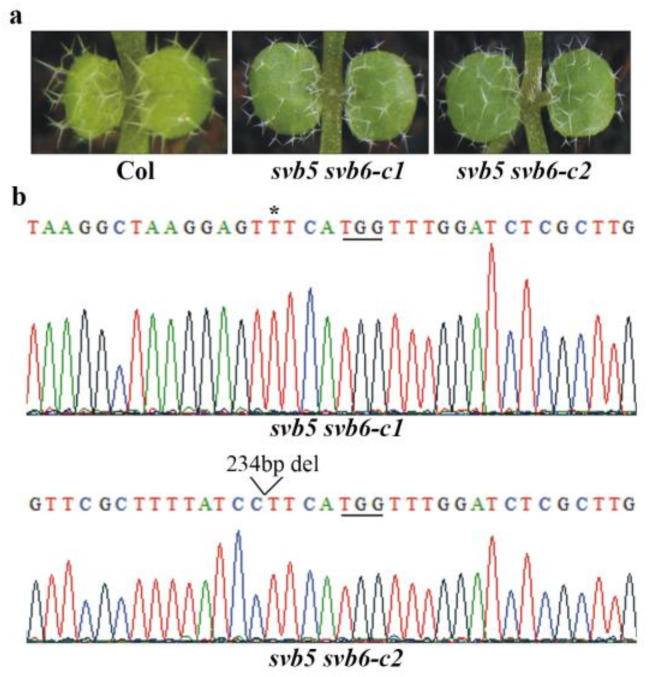
Mutation of *SVB5* and *SVB6* did not affect trichome development. (**a**) Trichome formation on the first two rosette leaves of the Col wild type, and the *svb5 svb6* double mutants. Seeds of the Col wild type, the *svb5 svb6-c1* and *svb5 svb6-c2* double mutants were sown directly into soil pots and grown in a growth room, and trichomes on the first two rosette leaves of 8-day-old seedlings were examine under a Motic K microscope and pictures were taken by using an EOS 1100D digital camera connected to the microscope. (**b**) Editing status of *SVB6* in the *svb5 svb6-c1* and *svb5 svb6-c2* double mutants. DNA was isolated from normal flowering T2 plants and used for sequencing. Underlines indicate the PAM sites, open arrow head indicates the site where a small fragments was deleted, and star indicates the single nucleotide insertion in the target sequence.

Since SVB3 is closely related to SVB4, and SVB5 is closely related to SVB6 (Figure 6a), we also generated *svb3 svb4* and *svb5 svb6* double mutants. We found that trichome formation was not affected in the *svb3 svb4* double mutants (Figure 7a). The *svb3 svb4* double mutants were generated by using a CRISPR/Cas9 construct targeting both *SVB3* and *SVB4* genes. In both of the *svb3 svb4* lines, a single nucleotide was inserted in the *SVB3* gene (Figure 7b). As for the *SVB4* gene, a single nucleotide was inserted for both lines, however, at different target sites (Figure 7c). Trichome formation in the *svb5 svb6* double mutants was also not affected (Figure 8a). The mutants were generated in the *svb5-c1* mutant background by editing *SVB6* gene, a single nucleotide was inserted in one line, and a 234bp fragment deletion was occurred in another line (Figure 8b).

**Figure 9:**
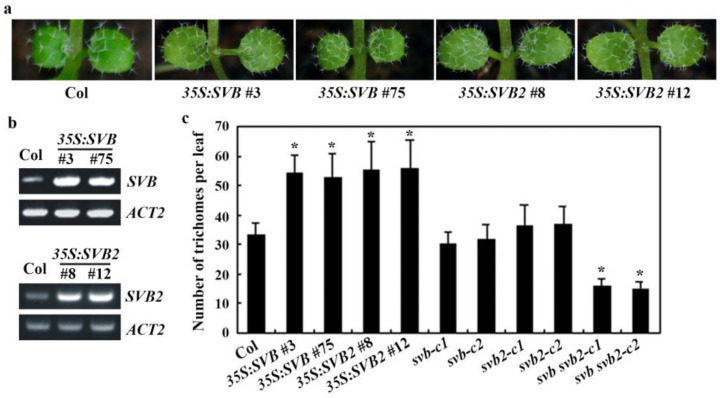
Overexpression of *SVB* and *SVB2* promote trichome formation. (**a**) Trichome formation in the Col wild type, the *35S:SVB* and *35S:SVB2* transgenic plants. Seeds of the Col wild type, the *35S:SVB* #3, *35S:SVB* #75, *35S:SVB2* #8 and *35S:SVB2* #12 transgenic plants were sown directly into soil pots and grown in a growth room. Trichome formation on the first two rosette leaves of 10-day-old seedlings were examined under a Motic K microscope and pictures were taken by using an EOS 1100D digital camera connected to the microscope. (**b**) Expression of *SVB* and *SVB2* in the *35S:SVB* and *35S:SVB2* transgenic plant seedlings, respectively. RNA was isolated from 12-day-old seedlings of the Col wild type, the *35S:SVB* and *35S:SVB2* transgenic plants, and RT-PCR was used to examine the expression of *SVB* and *SVB2*, respectively. The expression of *ACT2* was used as a control. (**c**) Trichome numbers on the first two rosette leaves of the Col wild type, the *35S:SVB* and *35S:SVB2* transgenic plants. Trichome formation on the first two rosette leaves of 10-day-old seedlings were counted under a Motic K microscope. Data represent the mean ± SD of 10 seedlings (20 leaves). * Significantly different from the Col wild type (*p* < 0.0001).

Since only *svb svb2* showed a trichome formation phenotype (Figure 6a), to further examine the functions of SVB and SVB2 in regulating trichome formation, we generated plants overexpressing *SVB* and *SVB2*, respectively. We found that trichome morphologies in the *35S:SVB* and *35S:SVB2* transgenic plants are largely similar to that of the Col wild type (Figure 9a), and RT-PCR results show that *SVB* and *SVB2* were overexpressed in the *35S:SVB* and *35S:SVB2* transgenic plants, respectively (Figure 9b). However, quantitative analysis show that both the *35S:SVB* and *35S:SVB2* transgenic plants produced more trichomes on the rosette leaves. Eventhough that in the *svb* and *svb2* single mutants remained largely unchanged when compare with the Col wild type, the trichome numbers on the rosette leaves of the *svb svb2* double mutants were only about half of that in the Col wild type (Figure 9c).

To examine how SVB and SVB2 may regulate trichome formation, we examined the expression of trichome formation key regulator genes in the *svb svb2* double mutants, we found that the expression of *EGL3* was increased when compared with that in the Col wild type seedlings (Figure 6d).

### 2.5. SVB and SVB2 Function Redundantly to Regulate Plant Growth and Development

**Figure 10:**
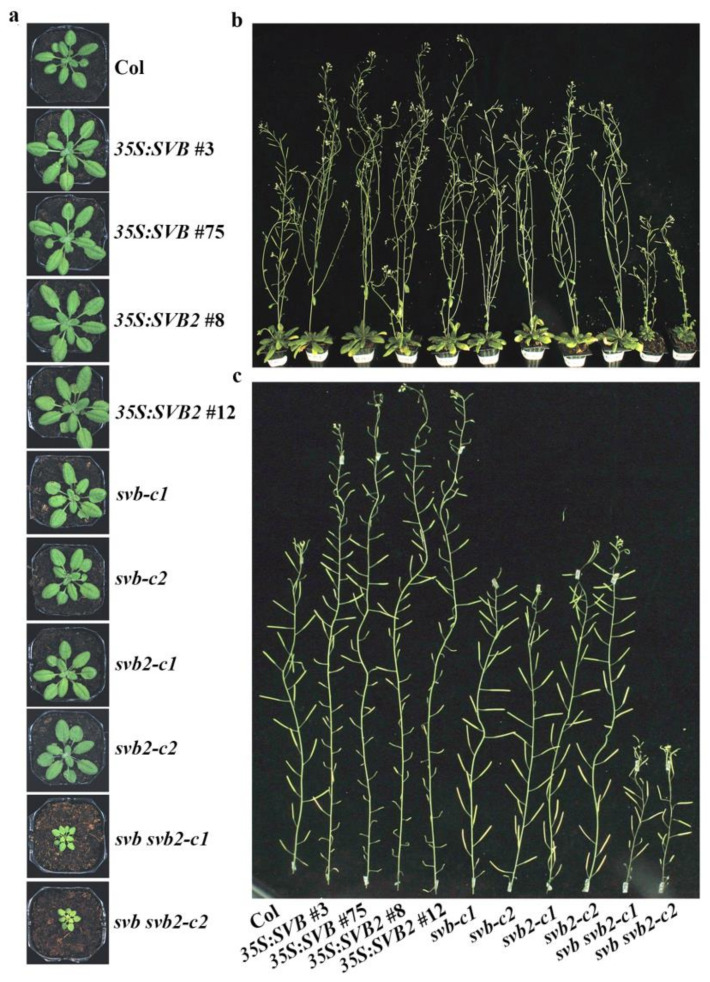
SVB and SVB2 function redundantly to regulate plant growth and development. (**a**) Four-week-old and (**b**) 7-week-old Col wild type, the *35S:SVB* and *35S:SVB2* transgenic plants, the *svb* and *svb2* single, and the *svb svb2* double mutants. (**c**) Close view of the main inflorescence stems of 7-week-old Col wild type, the *35S:SVB* and *35S:SVB2* transgenic plants, the *svb* and *svb2* single, and the *svb svb2* double mutants. Seeds of Col wild type, the *35S:SVB* and *35S:SVB2* transgenic plants, the *svb* and *svb2* single, and the *svb svb2* double mutants were sown directly into soil pots and grown in a growth room. Pictures were taken by using an EOS 1100D digital camera.

In addition to trichome formation, we found that SVB and SVB2 also function redundantly to regulate plant growth and development in Arabidopsis. As shown in Figure 10a, both the *35S:SVB* and *35S:SVB2* transgenic plants produced bigger rosettes when compared with that of the Col wild type. On the other hand, the rosette size of the *svb* and *svb2* single mutants is largely indistinguishable from that of the Col wild type, however, the *svb svb2* double mutants produced much smaller rosettes.

When reached mature stage, the plant height of the *35S:SVB* and *35S:SVB2* transgenic plants is higher than the Col wild type, whereas that of the *svb* and *svb2* single mutants is largely similar to the Col wild type, but that the *svb svb2* double mutants is much shorter than the Col wild type (Figure 10b).

We also noted that fertility of the *35S:SVB* and *35S:SVB2* transgenic plants was reduced, but that of the *svb* and *svb2* single mutants and the *svb svb2* double mutants is largely unaffected (Figure 10c).

## 3. Discussion

The DUF538 domain protein SVB has previously been identified as a regulator of trichome morphology, and its T-DNA insertion mutant *svb* plant produced variable branched small trichomes [47,48]. We provide evidence here that SVB and SVB2 function redundantly to regulate trichome formation in Arabidopsis.

Firstly, the gene edited *svb svb2* double mutants produced less trichomes (Figure 6, Figure 9), even though trichome numbers in the *svb* and *svb2* single mutants were indistinguishable from the Col wild type (Figure 9). The gene edited *svb* single mutants produced variable branched small trichomes (Figure 3), similar to previously reported for the T-DNA insertion *svb* mutants [47], indicating that the gene edited *svb* single mutants are loss-of-function mutants. Whereas gene edited *svb2* mutants are morphologically similar the wild type, but the *svb svb2* double mutants produced less trichomes (Figure 6, Figure 9), and the overall growth and development was also affected in the double mutants (Figure 10), suggesting that the phenotypes observed in the *svb svb2* double mutants are indeed caused by loss-of-function of both *SVB* and *SVB2* genes. Secondly, transgenic plant overexpressing *SVB* or *SVB2* produced more trichomes (Figure 9). Thirdly, the expression of the trichome formation key regulator gene *EGL3* was affected in the *svb svb2* double mutants (Figure 6).

It is well known that trichome formation in Arabidopsis is regulated by a few key regulators including TTG1, GL1, GL3/EGL3, GL2 and the R3 MYB transcription factor [3,4,5,6,7]. Some other regulators such as GIS proteins GIS and GIS3, ZINC FINGER proteins ZFP5 and ZFP8, SPL protein SPL9, and NAC protein NTL8, and several TCP proteins are also involved in the regulation of trichome formation either via regulating the expression of the MBW complex component genes [25,26,27,28,29,30,31,32], or affecting the formation or activation of the MBW complex [33]. We show that SVB and SVB2 function redundantly to regulate trichome formation in Arabidopsis (Figure 6 and Figure 9), and we found that the expression of *EGL3* is affected in the *svb svb2* double mutants (Figure 6). These results suggest that SVB and SVB2 may also regulate trichome formation via modulating the expression of trichome formation key regulator genes. However, the expression level of *EGL3* is increased in the *svb svb2* double mutants, whereas trichome formation is inhibited in the *svb svb2* double mutants (Figure 6), considering that EGL3 is a positive regulator of trichome formation [11], how SVB and SVB2 may regulate trichome formation still remained unclear. It is possible that increased expression level of *EGL3* may affected the formation of the MBW complex. However, since SVB and SVB2 also have redundant functions in regulating plant growth and development (Figure 10), and SVB has been found to bind PI(3)P and PI(3,5)P2 [49], and also plays a role in ER stress tolerance [48], it is also possible that SVB and SVB2 may use different mechanisms to regulate trichome formation in Arabidopsis.

Several plant hormones including GA, CTK and JA have been shown to be involved in the regulation of trichome formation in Arabidopsis, but in different ways. GA regulates the expression of *ZFP6*, and CTK regulates the expression of *ZFP8* and *GIS2*, which are able to regulate trichome formation via affecting the expression of trichome formation key regulator genes [25,26,27,29], whereas the JA signaling regulators JAZ proteins are able to directly interact with trichome formation key regulators GL1, GL3 and EGL3 [36]. Since *SVB* and *SVB2* are ABA response genes (Figure 1), the involvement of SVB and SVB2 in the regulation of trichome formation suggests that ABA may also involve in the regulation of trichome formation in Arabidopsis. To examine if defects in ABA biosynthesis or ABA signaling may affect SVB and SVB2 regulated trichome formation, and to identify the regulators of *SVB* and *SVB2* and examine their roles in trichome foramtion may help to reveal the roles of ABA in regulating trichome formation in Arabidopsis.

Considering that all the SVBs shared high amino acid identity and similarity, and at least the expression of *SVB5* and *SVB6* was also regulated by ABA (Figure 1), eventhough trichome formation was largely unaffected in the *svb3 svb4* and *svb5 svb6* double mutants (Figure 7 and Figure 8), it is still worthwhile to figure out if other SVBs may also be involved in the regulation of trichome formation by generating and characterizing *SVBs* high order mutants. On the other hand, at least *SVB5* and *SVB6* have different expression patterns with other *SVBs*, especially *SVB6* is predominantly expressed in flowers (Figure 2), and at least the subcellular localization of SVB4 and SVB6 are different from the other SVBs, it is possible that the SVBs may also have different functions. Generation of high order mutants may help to reveal the functions of SVBs in Arabidopsis.

In summary, we found that both *SVB* and *SVB2* are ABA response genes, and that SVB and SVB2 function redundantly to regulate trichome formation in Arabidopsis.

## 4. Materials and Methods

### 4.1. Plant Materials and Growth Conditions

The Columbia-0 (Col) wild type Arabidopsis was used for ABA treatment, protoplasts isolation, and plant transformation. The Ler wild type Arabidopsis was used as a control for examining the expression of *SVB* genes in the ABA biosynthesis mutant, *aba1-5* [51].

For ABA treatment and gene expression analysis, seeds of the Col and Ler wild types, the *aba1-5* mutant, the *SVBs* overexpression plants, and the *svbs* mutants were bleach sterilized, washed with sterilized water, and then plated on 0.6% (w/v) phytoagar (PlantMedia, Dublin, OH, USA) solidified, 1% (w/v) sucrose-containing ½ Murashige & Skoog (MS) plates. The plates were kept for 2 days in darkness at 4 °C, and then transferred to a growth room. For plant transformation and protoplast isolation, seeds of the Col wild type and *svbs* single mutants were sown into soil pots directly and grew in a growth room.

The temperature in the growth chamber was set at 22 °C, the photoperiod was at 16 h light/8 h dark, and the light density was at ~125 μmol m^−2^ s^−1^.

### 4.2. RNA Isolation and RT-PCR 

To examine the expression of *SVBs* in response to ABA, seedlings of 12-day-old Col wild type Arabidopsis were treated with 50 μM ABA for 4 h, and then samples were collected. To examine the expression of *SVBs* in *aba1-5* mutants, 12-day-old Ler wild type and *aba1-5* mutant seedlings were collected. To examine the expression pattern of *SVBs*, tissues and organs were collected from soil growing Col wild type Arabidopsis plants. To examine the expression of trichome formation key regulator genes, 12-day-old Col wild type, *SVBs* overexpression plants, and *svbs* gene edited mutants were collected. All the samples were frozen in liquid N_2_ immediately after collected, and then kept at −80 °C for RNA isolation.

Total RNA was isolated from the samples by using an EasyPure Plant RNA Kit (TransGene Biotech, Beijing, China), and cDNA was synthesized by using the EazyScript First-Strand DNA Synthesis Super Mix (TransGen Biotech), and by following the manufacturer’s instructions. RT-PCR was used to examine the expression of *SVBs* and trichome formation core regulator genes, as the basal expression levels of some genes, or gene expression levels in some tissues and organs are very low and are not suitable for qRT-PCR analysis. The primers used for RT-PCR analysis of *SVBs* are listed in Table S1, the primers for RT-PCR analysis of the trichome formation key regulator genes, and the *ACT2* control gene have been described previously [19,20,52].

### 4.3. Constructs

To generate *GFP-SVBs* constructs for protoplast transfection, the full-length open reading frame (ORF) sequences of *SVBs* were RT-PCR amplified by using RNA isolated from 12-day-old Col wild type seedlings, and cloned in frame with an N-terminal GFP tag into the *pUC19* vector under the control of the *CaMV 35S* promoter as described previously [53,54].

To generate *35S:SVBs* constructs for plant transformation, the amplified full-length ORF sequences of *SVBs* were cloned in frame with an N-terminal HA tag into *pUC19* vector under the control of the *CaMV 35S* promoter [53,54]. The *35S:SVBs* in the *pUC19* construct were then subcloned into the binary vector *pPZP211* [55].

To generate CRISPR/Cas9 constructs for gene editing of *SVBs*, potential target sequences were identified by scanning the exon sequences of *SVBs* on CRISPRscan (http://www.crisprscan.org/?page=sequence, accessed on 15 March 2017), and then evaluated on Cas-OFFinder (http://www.rgenome.net/cas-offinder/, accessed on 15 March 2017). The specific target sequences selected for editing *SVB* were 5′-CGCCACCGAGGTCATTGCAC(AGG)-3′ and 5′-GGACACCAACTGGTCTGTCC(AGG)-3′, for *SVB2* were 5′-GTTGGGTATGACAGAGAGTC(AGG)-3′ and 5′-GCTCACTGGAGTCAAAGCCA(AGG)-3′, for *SVB3* were 5′-GAAGGAGCAGAGATCTGCAA(TGG)-3′ and 5′-GAGCAAAGAGATTTTGATTT(GGG)-3′, for *SVB4* were 5′-GCTCTCATCAAACTACCCAC(GGG)3′ and 5′-GGAGATAACTGCGTTTGTTG(AGG)-3′, for *SVB5* were 5′-TGAGATCGTGTACGGGG(CGG)-3′ and 5′-GGCAGGTCTTTCCCCGTTAC(CGG)-3′, for *SVB6* were 5′-GGTTCGCTTTTATCCGAAAT(CGG)-3′ and 5′-GGTTAAGGCTAAGGAGTTCA(TGG)-3′. The target sequences were inserted into the *FT* expression cassette-containing *pHEE401E* vector as described previously [56]. The primers used for making *SVBs* gene editing constructs are listed in Table S1.

### 4.4. Plant Transformation and Transgenic Plants Selection

Transgenic plants were generated by using floral dip method [57], and Arabidopsis plants about 5-week-old when the main inflorescences have produced several mature flowers were used for transformation. The Col wild type Arabidopsis were transformed to generate overexpression plants, gene edited *svbs* single mutants and *svb3 svb4* double mutants. The *svb-c2* and *svb5-c1* single mutants were used to generate gene edited *svb svb2* and *svb5 svb6* double mutants, respectively. T1 seeds collected from the transformed plants were plated on ½ MS plates containing 50 μg/mL carbenicillin and 50 μg/mL kanamycin to select transgenic plants.

To obtain overexpression plants, T2 seeds collected from T1 transgenic plants were selected on ½ MS plates containing 30 μg/mL kanamycin to select lines with 3:1 segregation, and T3 seeds collected from T2 plants were selected ½ MS plates containing 30 μg/mL kanamycin for homozygous lines. Expression of *SVBs* in the transgenic plants was examined to obtain *SVBs* overexpression plants.

Gene edited Cas9-free *svbs* mutants were obtained by following the procedure described previously [56]. Briefly, gene edited status in T1 plants with early flowering phenotypes was examined, and T2 seeds collected from gene edited T1 plants were sown directly into soil pots, and gene edited status in the normal flowering (Cas9-free) T2 plants was examined, and Cas9-free status was further confirmed by amplifying Cas9 fragment in the mutants.

### 4.5. DNA Isolation and PCR

To examine gene editing status of *SVBs*, leaves of early flowering T1 transgenic plants or normal flowering T2 progeny of the gene edited early flowering T1 plants were collected, DNA was isolated and used for PCR amplification of *SVBs* genome sequences. PCR products were isolated and sequenced, and sequences obtained were aligned with the corresponding wild type *SVBs* sequences. DNA isolated from homozygous gene edited mutants identified from normal flowering T2 plants was also subjected to PCR amplification of the *Cas9* fragement to confirm the Cas9-free status. The primers used for amplifying *SVBs* genome sequences are listed in Table S1, and the primers used for amplifying *Cas9* fragment have been described previously [58].

### 4.6. Plasmid DNA Isolation, Protoplast Isolation and Transfection

Plasmid DNA of the *GFP-SVBs* constructs was isolated by using a GoldHi EndoFree Plasmid Maxi Kit (Kangwei, Beijing, China), and by following the manufacture’s procedure. Protoplasts were isolated from rosette leaves of ~4-week-old Col wild-type Arabidopsis plants, transfected with plasmid DNA of the *GFP-SVBs* constructs, and incubated in darkness at room temperature as described previously [41,54,59,60].

### 4.7. Morphological Assays

For morphological assays, seeds of the Col wild type, the *35S:SVBs* overexpression, single and double *svbs* mutant lines were germinated and grown in soil pots. Pictures of the plants were taken at indicated growth stages by using a digital camera.

### 4.8. Microscopy

Trichome phenotypes of 8- or 10-day-old Col wild type, the *SVBs* overexpression plants and the *svbs* mutants were examined, and pictures were taken under a Motic K microscope which was equipped with an EOS 1100D digital camera. Trichome numbers on the first true leaves was calculated, and statistical analysis was performed by using Student’s t-Test (https://www.graphpad.com/quickcalcs/ttest1/?format=SD, accessed on 17 June 2021). GFP fluorescence in the transfected Arabidopsis protoplasts was examined, and pictures were taken under an Olympus FV1000 confocal microscope.

## Data Availability

All data obtained were presented in this article.

## Supplementary Materials

The Supplementary Materials are available online at https://www.mdpi.com/article/10.3390/ijms22136790/s1.
