# Supplementary material for: Involvement of ABA Responsive SVB Genes in the Regulation of Trichome Formation in Arabidopsis
Source: Int J Mol Sci. 2021 Jun 24;22(13):6790. doi: 10.3390/ijms22136790 (PMC8268597; doi:10.3390/ijms22136790)
Supplement: Supplementary file 1 [file ijms-22-06790-s001.zip › ijms-1259360-supplementary.pdf]

Table S1. Primers used in this study.

Primers	Sequences (5′-3′)
SVB-F-NdeI	CAACATATGGGTTTGGTTACAGATGAAGT
SVB-R-SacI	CAAGAGCTCCTAAGCATCTGTGACTGCAAC
SVB2-F-Nde1	CAACATATGGGTTTGGTTACAGAGG
SVB2-R-Sac1	CAAGAGCTCTTAGGCCGCCTCTTTGA
SVB3-F-Nde1	CAACATATGTCGTCTCAAGAAACGAA
SVB3-R-Sac1	CAAGAGCTCTCATTCGTCTTCTTCAAAAGC
SVB4-F-Nde1	CAACATATGGCGTTAAGAGAGATGATA
SVB4-R-AFlll	CAACTTAAGTTACATCAAGGAAGTCTGGTTAC
SVB5-F-NdeI	CAACATATGGCGAGCGAGGGAGTTGT
SVB5-R-AflII	CAACTTAAGTTAGTTTTTGGGATTCTCCAA
SVB6-F-Nde1	CAACATATGGTGACAGAAGCCATG
SVB6-R-Sac1	CAAGAGCTCTTAGTACAAATAGATTTTGGTTTTG
DT1-BsF (*SVB*)	ATATATGGTCTCGATTCGCCACCGAGGTCATTGCACGTT
DT1-F0 (*SVB*)	TCGCCACCGAGGTCATTGCACGTTTTAGAGCTAGAAATAGC
DT2-R0 (*SVB*)	AACGGACAGACCAGTTGGTGTCCAATCTCTTAGTCGACTCTAC
DT2-BsR (*SVB*)	ATTATTGGTCTCGAAACGGACAGACCAGTTGGTGTCCAA
DT1-BsF (*SVB2*)	ATATATGGTCTCGATTGTTGGGTATGACAGAGAGTCGTT
DT1-F0 (*SVB2*)	TGTTGGGTATGACAGAGAGTCGTTTTAGAGCTAGAAATAGC
DT2-R0 (*SVB2*)	AACTGGCTTTGACTCCAGTGAGCAATCTCTTAGTCGACTCTAC
DT2-BsR (*SVB2*)	ATTATTGGTCTCGAAACTGGCTTTGACTCCAGTGAGCAA
DT1-BsF (*SVB3*)	ATATATGGTCTCGATTGAAGGAGCAGAGATCTGCAAGTT
DT1-F0 (*SVB3*)	TGAAGGAGCAGAGATCTGCAAGTTTTAGAGCTAGAAATAGC
DT2-R0 (*SVB3*)	AACAAATCAAAATCTCTTTGCTCAATCTCTTAGTCGACTCTAC
DT2-BsR (*SVB3*)	ATTATTGGTCTCGAAACAAATCAAAATCTCTTTGCTCAA
DT1-BsF (*SVB4*)	ATATATGGTCTCGATTGCTCTCATCAAACTACCCACGTT
DT1-F0 (*SVB4*)	TGCTCTCATCAAACTACCCACGTTTTAGAGCTAGAAATAGC
DT2-R0 (*SVB4*)	AACCAACAAACGCAGTTATCTCCAATCTCTTAGTCGACTCTAC
DT2-BsR (*SVB4*)	ATTATTGGTCTCGAAACCAACAAACGCAGTTATCTCCAA
DT1-BsF (*SVB5*)	ATATATGGTCTCGATTGGAGATCGTGTACGGGGCGGGTT
DT1-F0 (*SVB5*)	TGGAGATCGTGTACGGGGCGGGTTTTAGAGCTAGAAATAGC
DT2-R0 (*SVB5*)	AACGTAACGGGGAAAGACCTGCCAATCTCTTAGTCGACTCTAC
DT2-BsR (*SVB5*)	ATTATTGGTCTCGAAACGTAACGGGGAAAGACCTGCCAA
DT1-BsF (*SVB6*)	ATATATGGTCTCGATTGGTTCGCTTTTATCCGAAATGTT
DT1-F0 (*SVB6*)	TGGTTCGCTTTTATCCGAAATGTTTTAGAGCTAGAAATAGC
DT2-R0 (*SVB6*)	AACTGAACTCCTTAGCCTTAACCAATCTCTTAGTCGACTCTAC
DT2-BsR (*SVB6*)	ATTATTGGTCTCGAAACTGAACTCCTTAGCCTTAACCAA
SVB3-TG1-gRT#+	AAGGAGCAGAGATCTGCAAGTTTTAGAGCTAGAAAT
SVB3-TG1-AtU6-29T#-	TTGCAGATCTCTGCTCCTTCAATCTCTTAGTCGACT
SVB3-TG2-gRT#+	AGCAAAGAGATTTTGATTTGTTTTAGAGCTAGAAAT
SVB3-TG2-AtU6-1T#-	AAATCAAAATCTCTTTGCTCAATCACTACTTCGTCT
SVB4-TG1- gRT#+	CTCTCATCAAACTACCCACGTTTTAGAGCTAGAAAT
SVB4-TG1-AtU6-1T#-	GTGGGTAGTTTGATGAGAGCAATCACTACTTCGTCT
SVB4-TG2- gRT #+	GAGATAACTGCGTTTGTTGGTTTTAGAGCTAGAAAT
SVB4-TG2- AtU6-26T#-	CAACAAACGCAGTTATCTCCAATCACTACTTCGACTC
